# The Work of Micro-Organisms

**Published:** 1887-06

**Authors:** Hiram Christopher

**Affiliations:** Missouri


### THE WORK OF MICRO-ORGANISMS.

BY HIRAM CHRISTOPHER, M. D., OF MISSOURI.

At no time previous has there been a greater interest shown for bacterial investigation than that which has been so apparent and universal during the last few years; and though this interest, in a great measure, may have been excited by the desire to establish favorite theories or confirm preconceived opinions, still many facts have been gathered which at some future time may form material for a more rational hypothesis respecting the action of micro-organisms than any yet presented, and particularly as respects their connection with abnormal processes observed in both plants and animals. In this paper I propose to offer a few reflections on the action of these structures in general, and to discover, if possible, what relation micro-organisms sustain in the living world, and what connection they hold to morbid action, whether as cause or effect of such action. The discussion will be conducted mostly on general principles as affording the safest protection against self-deception.

By micro-organisms we mean those microscopic structures whose germs or spores exist so abundantly in the air, by whatever name called, whether germ, spore or egg, the *punctum saliens* of the perfected structure. They conform essentially to the universal law of the organic world, and have their analogues in the fecundated cell of the seed of plants and ova of animals. Originally they came into being by an act of creative power, and are perpetuated by the system of reproduction. Hence *omne vivum ab vivo* expresses the fundamental truth of the organic world. To this fundamental and universal law there is no exception; and whatever may be the form which the germ may present, whether a simple isolated cell, or whether imbeded in pabulum, as in the seed of phenogamous plants, or whether connected with parent during development, the fertilized cell is essentially the same in all living organisms of the organic kingdom.

All initial cells of new individuals arise from specialized cells of different sexes in which they have attained this specific character by differentation. In highly complex organisms, we have various kinds of cells, as respects structure and function, arising from a common initial point, the elements of special organs with specific function; but however the living cells of distinct organs may differ, they yet have the same essential elements of structure, namely, living matter, isolated by an investing membrane, and thus made individual. We may, therefore, contemplate the highest and most complex organisms as but an aggregation of living cells, microscopical as to dimensions, and are analogous to individual micro-organisms. The two classes differ only in form and function. Could the differentiated cells of complex organisms, isolated and made to appear as individual organisms, the same as the infusoria, for instance, the analogy would become identity.

Cell-organisms of all kinds are essentially the same, in being encysted living matter. This matter is called living because it manifests vital phenomena. These are essentially and primarily motion, growth and reproduction, to which may be added differentiation. In the operation of these functions the life of species is continued on the earth, and all the various grades of structure and function are developed and maintained, while the basis of all vital operations lies in motion, without which life ceases. The primary work, therefore, of all living organisms is to ingest and assimilate food, that they may grow and reproduce. Such a work is constructive.

In the constructive work of life, there is, however, a destructive work. Construction follows immediately on the assimilation of pabulum, and destruction is a necessary attendant on the constructive process. The construction is from pabulum, and destruction comes of the use of the pabulum in the process of nutrition. The former gives us organic and living fabric; the latter, excretory substances arising from the operations of living cells in the discharge of their functions. These excretions are both organic and inorganic, as respects chemical constitution, a fact that shows that the nutritive process is the means by which organic matter of the pabulum in part is sent toward and back into the realm whence it was originally taken by the chlorophyl cells of plants. The process of food-making by these cells, which is a little better understood than food-assimilation, affords us some conception of the chemical changes in pabulum that take place in nutrition. When the elements of food, carbon dioxide, ammonia, in the main, dissolved in water, reach the chlorophyl cells of plants, found mostly in their leaves, these inorganic compounds undergo decomposition, on or during which new combinations are formed by which the substance called elaborated sap is produced, which serves the plant as food. In the chemical changes which take place in the preparation of the elaborated sap, oxygen is set free. It is the residuum of the chemical changes that take place in the living matter of the food-making cells. So, in the nutrition of animals, carbon dioxide and water are two inorganic compounds that result from the changes that take place in the assimilation of food by the living cells of all kinds in complex organisms. These are excretions of inorganic structure, besides which there are also excretions of substances which are organic as respects their chemical constitution of a low grade of structure and unfit for any use in the organism, and more or less poisonous to its living matter. But such excretory substances may be, and in most instances are, nutritious pabulum for other organisms, as when voided urine swarms with bacteria or other micro-organisms. It is to be particularly noted, however, that in the excretions of all organisms, whether small or great, a part is inorganic matter, and a part organic of a constantly descending grade of structure, until it reaches a composition which is no longer susceptible of nutritive action, as are the carbohydrogens, so abundantly generated in plants.

Now as all changes which pabulum undergoes in complex organisms, are produced during the process of nutrition going on in the vital units of the organism, we have but to contemplate these living cells as micro-organisms to understand what the work of these is, and what are the results of their work. When we subject an infusion of animal matter to their action, we find that odorous gases are produced, some of which are inorganic as respects their composition; a film also covers the fluid, and this is organic matter; but both gas and film are excretory substances. After an exposure of several days, different odors will appear, and new organisms. So if we take some surface water from a pond covered by a scum, we will find certain organisms when first examined, and afterward other kinds. A few days are sufficient to exhibit these phenomena; but these exhibits will appear time after time in the same month. So, again, if such pools of water are examined during different months, or seasons, as in the spring, summer and fall, different species of organisms will be found characterizing the different months and seasons. These facts are cited simply to show that appearance or development of micro-organisms follows a universal law of the organic kingdom, which law is that temperature and food determine what species of micro-organisms will be developed or nourish in water in any particular locality. These conditions are the necessary antecedents and concomitants; but in no case the consequences of the appearance of micro-orgrnisms. When germs find the essential antecedents, they nourish and mature, and produce under like circumstances. Their excretions appear in the odorous gases and film which cover the surface of the fluid. These are in, or border on, the inorganic world, just as is observed in the excretions of the higher complex organisms. Their action, therefore, is essentially the same as that of the vital units of the latter.

Organic molecules differ from the inorganic, as repects their chemical constitution, in the relative position of atoms in the molecules. In the carbonate of ammonia $(NH_4)_2 CO_3$) we have all the elements of a nitrogenous compound, but there is no nutrition in it. The cyanate of ammonia has the same chemical constitution as urea; but we find that only urea is nutritious to micro-organisms. The inorganic salts are poisonous to them; and hence antiseptic. The difference between these and similar molecules lies in the relative position of their atoms in the molecules. This relative position makes the great difference. In the inorganic salts the position of the atoms is determined by chemism alone; in the organic nutritious molecules, by the same force, but dominated and directed by the vital. This difference is clearly seen in the action of the food-making cells of plants. Since, therefore, the vital force dominates the chemical in all organic compounds, and especially in those nearest the vital process, as the tissues of animals, organic substances are really more stable as repects chemical constitution, than are inorganic substances. This fact is stated here in order that the work of nutrition in the decomposition of organic compounds may be the more clearly apprehended and appreciated.

As the process of nutrition is the regularly established means of decomposing organic matter and of restoring its atomic elements to the realm whence they were taken by the food-making cells of plants; and as complex organisms effect but little, comparatively of this work, a great army of cormorants is spread everywhere, appointed to complete what was left undone by the higher forms, and, as the ultimate scavengers of the organic world, to consume the last vestige, and in their excretions to break up as far as this is possible, the organic reunion of atomic elements. Their work is the same as the higher, only they operate finally on the last vestiges of organic substances. They lie at the end of the long series of living organisms as we look downward; or at the beginning, as we look upward; and are intended, in the great work of Nature, to complete the work impossible of completion by the vital units of other and higher organisms, that work being the adjustment of the quantity of matter in the two kingdoms of Nature. Such an adjustment is necessary, since there is no loss of matter during the circuit it is made to take by the vital force from the world of dead matter upward through the varied and numerous forms of life, and then downward and back again to the inorganic world. As inorganic matter is continually converted into organic, the adjustment requires that organic matter shall be continually and as regularly returned to the inorganic state. This we find to be done by the regular operation of the laws of life. The vital agent composes and decomposes organic matter, since its power dominates chemical force in all organic compounds.

The organic remains of living organisms that once lived and flourished on the earth, are but the inorganic constituents of those once living beings. Their perishable parts disappeared, as do those of living creatures at the present day. In all ages the proper adjustment between the two realms of matter has been preserved, so that no age has encroached on its successor by its unconsumed lifeless fabrics, except in those cases which *design* has marked as exceptions, as in the vast coal fields that were laid down in anticipation of their use by man. We thus see that in every age of the earth's history the work of life has been conservative in the main, and destructive only that its constructive work might continue uninterrupted. Life builds and life destroys.

While such has certainly been the work of life on the earth, and such its design, it would seem strange and anamolous if we should find any great department of the life-kingdom, as the microscopic world, set apart to the special work of destruction, and this only, as their connection with morbid action, as the cause presumes them to be. Were they capable by their presence and normal action, in their normal growth and development, of producing diseased action in healthy organisms, micro-organisms would, on account of their countless number, be more destructive of life than would all the beasts of prey, if they were given access to the great fields of animal life. As a fact, however, they are capable of doing no more in this direction than any other grade of living beings, nor by any different means or methods. Their poisonous excretions are the analogues of the poisonous substances formed in the higher organisms, as the acids, alkaloids and natural substances found in plants and animals, the products of the action of their living cells. When, therefore, micro-organisms are found associated with morbid action, their relation to such action is not that of cause; but one arising from the nature of the pabulum produced from the structures of the organism suffering from disease by the morbid action, and the character of the pabulum will be changed and no longer suited to the nutrition of the organisms, and they will disappear. Illustrations of this are numerous throughout the organic kingdom. But these organisms, while rapidly multiplying in fluids that are nutritious, may, and possibly do, add to the morbid action by their excretions, just as urine in man will, by absorption, prove injurious or fatal.

These things being true there is not, and cannot be, such a thing as *a septic organism*. There are septic *substances*, and these in abundance. This is a matter familiar to every one. The saliva of a rabid dog, or the poisonous fluid of a venomous reptile is the product of a living organism, one laboring under a fatal disease, and the other in its normal state. So, as respects all animal and vegetable poisons, whatever the grade of the organism may be. These are all generated in the organisms, because they are mostly of an organic composition; and hence morbific agents may be organic *substances*, but not *living organisms*. After all the observations and experiments made to determine the relation of micro-organisms to morbid action, Klein says: "Amongst the legion of different species of micro-cocci and baccilli occurring in putrid substances, the great majority are quite harmless; when introduced into the body of an animal, they are unable to grow and multiply, and, therefore, unable to produce any disturbance." Some three or four species constitute the minority according to the same authority. And even when these few supposed septic organisms do occasion any disturbance in an organism, the same author further says that it is necessary to discriminate and "consider whether the relation of the specific micro-organism to the essence of the disease, or, in other words, the question whether the organism itself is the virus, or whether this latter is the product of the former. Something in the same way as the ferment is the product of the putrefactive organism." (Micro-Organisms and Disease, pp. 167, 180). This statement is a little cloudy from the use of ambiguous terms; but it is sufficiently so to lead us to understand that the author is not fully satisfied, and is not sure but that the excretory products of micro-organisms are the real virus or cause of morbid action.

Again: in regard to certain diseases which are supposed to be produced by septic germs, as "tubercle, glanders and swine-plague," in which the number of organisms is but few in comparison with the extent of the diseased surface, he further says that "the pathological condition brought about by the organisms is not due to the direct action of their numbers; but it is an indirect sequence brought about by definite chemical alterations in the blood or tissues, as the case may be." Respecting these "chemical alterations," he says that two theories may be assumed as possible:

1. "That these chemical effects are produced by the presence and growth of the organisms, as truly as in the alcoholic fermentation of sugar, the alcohol produced is the result of the presence of the saccharomycetes," and,

2. "That the organism elaborates a specific ferment which, after a certain amount has been produced, sets up the particular pathological changes."

If we are to understand "chemical alterations" and "chemical effects" as meaning the products of nutritive action going on in micro-organisms, then the virus, producing morbid action, is not the organism itself, but the excreta of the organism. These are not properly chemical, but vital products, since they are the product of the vital force in the process of nutrition. So, under both assumptions, it is the excretory product of nutrition, active in micro-organisms, and not their bodies, that gives rise to the "particular pathological changes." This is, indeed, his own final conclusion. "From these considerations," he says, "it follows that the virus cannot be considered independent of the organism; we cannot assume that the two can be separated from one another; for the most feasable assumption, and the one borne out by observation is, that, owing to the multiplication of the organisms, certain chemical changes are produced in the blood and tissues, or that a special ferment is created, which sets up the anatomical changes characteristic of the particular disease" (pp. 182–3). This is somewhat obscure, but its very obscurity shows that the author of a volume on "*Micro-Organisms and Diseases*" is not fully convinced in his own mind that there are *any septic organisms.*

Investigations of the phenomena attending the development of micro-organisms in solutions of organic matter have thus far thrown the weight of facts against the assumption of spontaneous generation. It is now well nigh universally admitted that living beings have their origin in creative power, and have been multiplied on the earth by a system of reproduction by which one individual arises from the organism of another. If such investigations had been attended by no other result than this, the microscope would have proved itself to be an inestimable aid to scientific inquiry. But more than this has been learned from its relations in regard to the relative action of the vital and chemical forces. The sum of this knowledge may be stated in the form of a simple proposition, namely: *that spontaneous decomposition of organic matter*, or its decomposition under the action of physical agents alone, *is not a law of Nature.* That is to say, that organic matter does not decompose under the ordinary operation of purely chemical agents, commonly so-called. Facts prove that organic matter is really more stable in composition than inorganic. This we would naturally expect from the dominancy of the vital force in all cases where it and the chemical act together. When once formed, the relations of the atoms in organic molecules, and their chemico-vital attraction, are not easily disturbed or broken. Even under the action of the vital force in the nutritive process, the descent of organic matter into the inorganic world is very slow and gradual. Sugar, for instance, an excretory product of the vital cells of plants, will remain unchanged forever if kept perfectly dry and free of albuminoid matter. Not until this and moisture are added will it undergo change as in fermentation. So with animal matter. Tyndall preserved infusions of this kind of matter hermetically sealed in glass tubes for a year without change. If flesh be kept *perfectly* dry it will remain unchanged. Moisture and an albuminous compound are not necessary to chemical decomposition; but they are to the processes of fermentation and putrefaction, because solution of organic matter in water is necessary to the growth of micro-organisms on it. In and by their nutrition, organic matter takes its course upward into living matter and then downward and onward into the realm of pure matter. This is true of all organic matter that is nutritious. The leaves of trees do not decompose; but are consumed by fungus. The tree itself passes away by the same means. Rotten wood is but granular fungus. Such are the revelations of the microscope. It has pointed out the true process of Nature. What was once considered chemical is now known to be vital. The microscope has been our instructor. Chemical changes in organic matter are now known to be vital changes. Such is the decision of modern researches. "These have shown that the decomposition of organic matter in the soil [or anywhere else] is due to the vital activity of living organisms, and that a variety of chemical products is formed while it is taking place, some of which are assimilated by the micro-organisms which are the active agents in the process of decomposition, and some of which are set free—(Sternburg). As respects the properties of these "chemical products" of vital action he further says: "We do know that certain complex nitrogenous substances produce most violent toxic effects in minute doses. As examples of this we may mention the septic poison-sepsin, which has been obtained by Bergmann, by Panum, by Burdon-Sanderson from putrid animal matter; the ptomaines, which resemble the vegetable alkaloids in their reactions, and which have been studied by Selmi and Gautier, who obtained them not only from putrid blood, but also from normal secretions of healthy persons; and proteids obtained by Weir, Mitchell and Reichert from serpent venom." To the same purport Klein speaks: "Gaspard, Panum, Billroth, Burdon-Sanderson, Gutman, and Semmer, and many others, have shown that by putrefaction of animal substances, a substance can be obtained—the septic

2

poison, or sepsin—which can be isolated by various chemical processes destructive of every living organism, and which on injection into the vascular system of animals, especially dogs, in sufficient quantities, produces marked febrile rise of temperature, and is capable of causing death with the symptoms of acute poisoning; e. g. shivering, vomiting and purging, spasms, torpor, collapse and death."

These extracts corroborate the fundamental position taken in this paper, viz: that morbific agents are the excreta of living organisms, whether these be micric or macric. Putrid blood from which ptomaines were obtained was made putrid by the ptomaines, the excreta of micro-organs; and the secretions of healthy persons furnished ptomaines only after micro-organisms had developed in them and excreted these poisonous substances.

Such are the revelations of the microscope; such its service to sicentific investigation. Its use has led to better and more correct views of the vital operations in nature. If its revelations are not misinterpreted, it will protect the observer from self-imposition, and lead him into the paths of fact and truth. *Cum hocs, post hocs*, and *propter hocs* will be assigned their true position in phenomena, and science will continue to unfold the mysteries of Nature unimpeded by mistaken or illogical conclusions.

